# Hepatic peroxisome proliferator-activated receptor γ and α-mRNA expression in HCV-infected adults is decreased by HIV co-infection and is also affected by ethnicity

**DOI:** 10.6061/clinics/2015(12)05

**Published:** 2015-12

**Authors:** Nathan J Shores, Maria Cássia Mendes-Corrêa, Ivana Maida, JoLyn Turner, Kevin P High, Sergio Babudieri, Marina Núñez

**Affiliations:** I Wake Forest University Health Sciences, Department of Internal Medicine, Section on Infectious Diseases, Medical Center Boulevard, Winston Salem, NC, United States..; II Faculdade de Medicina do ABC, Unidade de Referência em Doenças Infecciosas, Santo André/SP, Brazil.; III Hospital das Clínicas da Faculdade de Medicina da Universidade de São Paulo, Moléstias Infecciosas e Parasitárias, São Paulo/SP, Brazil.; IV Università degli Studi Sassari, Istituto Malattie Infettive e Parassitarie, Sassari/SS, Italy.; V Charleston Gastroenterology Spec, Charleston/SC, United States.

**Keywords:** Nuclear Receptors, PPAR, Hepatitis C, HIV Infection, mRNA Expression, Liver Tissue

## Abstract

**OBJECTIVE:**

To determine peroxisome proliferator activated receptor α and γ mRNA expression in liver tissue of hepatitis C virus-infected patients with and without human immunodeficiency virus and its possible contribution to an acceleration of liver disease progression.

**METHODS:**

We measured peroxisome proliferator-activated receptor α and γ mRNA expression by real-time polymerase chain reaction in liver tissues from 40 subjects infected only with hepatitis C virus, 36 subjects co-infected with hepatitis C virus and human immunodeficiency virus and 11 normal adults.

**RESULTS:**

Hepatic mRNA expression of both peroxisome proliferator-activated receptors was significantly lower in hepatitis C virus-infected subjects with and without human immunodeficiency virus co-infection compared to the controls. Non-black race was also identified as a predictor of lower peroxisome receptor α and γ mRNA expression. Compared to subjects infected only with hepatitis C virus, liver peroxisome receptor γ mRNA expression was significantly lower in hepatitis C virus/human immunodeficiency virus-co-infected subjects (0.0092 in hepatitis C virus/human immunodeficiency virus-co-infection *vs*. 0.0120 in hepatitis C virus-only; *p*=0.004). Hepatic peroxisome receptor α mRNA expression in the hepatitis C virus-infected patients was lower in the presence of human immunodeficiency virus co-infection in non-black subjects (0.0769 *vs*. 0.1061; *p*=0.02), whereas the levels did not vary based on human immunodeficiency virus status among black subjects.

**CONCLUSION:**

mRNA expression of both peroxisome proliferator-activated receptors is impaired in hepatitis C virus-infected liver and further reduced by human immunodeficiency virus co-infection, although the suppressive effects of the viruses are substantially mitigated in black patients.

## INTRODUCTION

Chronic hepatitis C virus (HCV) infection in the setting of human immunodeficiency virus (HIV) co-infection is characterized by accelerated liver disease, although the underlying reasons are unclear 1. Activation of peroxisome proliferator-activated receptor (PPAR) α and γ, members of the nuclear receptor superfamily, inhibits the expression of inflammatory cytokines and chemokines and reduces hepatic inflammatory processes 2. Additionally, PPARγ activation maintains a quiescent hepatic stellate cell phenotype and decreases pro-fibrogenic products 2. The HCV core protein reduces the expression of hepatic PPARα in HCV mono-infected patients 3. The role of PPARγ in the pathogenesis of HCV-related liver disease is less clear because the data are conflicting 2,5. Decreased expression of PPARγ mRNA has been reported in liver biopsies of HIV-infected subjects with insulin resistance and unexplained liver enzyme elevation 6, suggesting an effect of HIV on liver PPARγ expression. This result is supported by several *in vitro* and *in vivo* studies that reported evidence for HIV infection of the liver 7.

We hypothesized that HIV infection reduces the expression of PPARα in the HCV-infected liver. We also hypothesized that reduced PPARα and PPARγ expression would accelerate the process of liver fibrosis in patients with HIV/HCV co-infection.

## METHODS

We conducted a multicenter, comparative, cross-sectional study that included liver tissue samples from HCV-only infected, HCV/HIV-co-infected and HCV/HIV-uninfected control subjects. The local institutional review boards (IRB) approved the research project. Informed consent was obtained from each patient included in the study and the study protocol conformed to the ethical guidelines of the 1975 Declaration of Helsinki as reflected in an *a priori* approval by the institution's human research committee. The participating centers were Wake Forest University Health Sciences, Winston Salem, NC, USA, Unidade de Referência em Doenças Infecciosas, Faculdade de Medicina do ABC, Santo André, São Paulo, Brazil, Hospital das Clínicas, Faculdade de Medicina da Universidade de São Paulo, São Paulo, Brazil, and Istituto Malattie Infettive, University of Sassari, Sassari, Italy.

### Selection of subjects

Subjects chronically infected with HCV, with or without concurrent HIV co-infection, who were 18 years of age or older were eligible for the study. Active HCV or HIV infection was defined as detectable plasma HCV RNA or a positive anti-HIV antibody titer at the time of enrollment, respectively. Subjects were excluded if other causes of liver disease were present, including hepatitis B virus (HBV) co-infection (HBsAg positive), hemochromatosis, autoimmune hepatitis, Wilson’s disease, or alpha1-antitrypsin deficiency. Alcohol intake was not an exclusion criterion, but the amount consumed was specified as accurately as possible based on patient reports. Liver samples from 11 HIV- and HCV-uninfected control subjects that were previously collected as part of another IRB-approved study at Wake Forest University Health Sciences were used for comparative analysis. At the time of collection, the control subjects had screened negative for other known liver diseases. However, no clinical or demographic data were available for these subjects.

### Data collection

Data were collected from medical records and included age, sex, weight, body mass index (BMI; kg/M^2^), race, country of origin, HCV genotype, serum HCV RNA, alanine aminotransferase (ALT) and aspartate aminotransferase (AST) levels, liver biopsy data (fibrosis, inflammation, and steatosis), absolute and % CD4+ lymphocytes, HIV RNA serum levels, highly active antiretroviral therapy (HAART) at the time of liver biopsy, diabetes mellitus (DM) status, alcohol intake, and serum cholesterol and triglyceride levels. Data from liver biopsies included METAVIR scores for fibrosis (F0 = no fibrosis, F1 = portal fibrosis without septa, F2 = portal fibrosis with few septa, F3 = numerous septa without cirrhosis, and F4 = cirrhosis), inflammation (‘activity score’ of A0 = no activity, A1 = mild activity, A2 = moderate activity, and A3 = severe activity), and steatosis scored as used in non-alcoholic fatty liver disease (<5%, none, 5-33%, mild, 34-66%, moderate, and >66%, severe).

### Tissue Preparation

All HCV-infected or HCV/HIV-co-infected subjects underwent liver biopsies as part of the routine medical care for their liver disease according to local practices. A portion of each biopsy was stored immediately in RNA stabilizing solution (RNAlater, Ambion, Austin, TX, USA) according to the package insert. Total RNA was extracted from the liver biopsies using RNA STAT-60 Reagent (Tel-Test, Friendswood, TX, USA) according to the manufacturer’s protocol. Briefly, liver tissues were homogenized in RNA STAT-60. Then, chloroform was added and the samples were centrifuged to separate the homogenate into two phases. The upper aqueous phase was transferred to a nuclease-free tube and mixed with isopropanol, and the RNA was precipitated overnight at -20°C. After centrifugation, the RNA pellet was washed with 75% ethanol, briefly air dried and re-suspended in nuclease-free water. The quality of the RNA was confirmed using the Agilent Bioanalyzer system.

### Quantification of PPARα and PPARγ mRNA expression by RT-PCR

Two micrograms of RNA was reverse-transcribed to cDNA using the Applied Biosystems High Capacity RNA to cDNA Kit (Applied Biosystems, Foster City, CA, USA). PPARα, PPARγ, and GAPDH expression was assessed by real-time PCR (RT-PCR) using pre-formulated TaqMan Gene Expression Assays for each gene (Applied Biosystems, Foster City, CA, USA) composed of two unlabeled primers and a FAM dye-labeled MGB probe designed to span introns and avoid the amplification of genomic DNA. The RT-PCR results were calculated using the comparative Ct method based on relative gene expression compared to GAPDH.

### Statistical methods

The primary outcome of the study was the analysis of comparative mRNA expression of PPARα and PPARγ in the livers of HCV-only *versus* HCV/HIV-co-infected subjects. Based on a previous report in HCV-infected subjects in which the mean PPARα level was 1.8 and the standard deviation was 2.8 7, 39 individuals were needed in each group to rule out equivalence of the means (HCV and HCV/HIV groups) and to obtain a desired power of 80% and two-sided significance level of 5%. Eleven samples from subjects negative for viral hepatitis were included for comparison (control group).

The results are presented in absolute numbers for continuous variables and in proportions for categorical variables. Comparisons between groups were performed using Fisher’s exact test for categorical variables and the t or ANOVA test for continuous variables. Univariate and multivariate linear regression analyses were performed to identify independent outcome predictor factors of PPARα and PPARγ. All *p*-values <0.05 were considered statistically significant. Statistical Package for the Social Sciences (SPSS), version 20.0 (SPSS Inc, Chicago, IL, USA) was used for statistical analysis.

## RESULTS

We were able to obtain adequate liver samples for the study from 40 HCV-only infected and 36 HCV/HIV-co-infected subjects. The characteristics of the subjects are summarized in Table 1. Demographics were similar between the two groups with the exception of race and country of origin. There were more white subjects in the HCV-only infected group. Serum HCV RNA levels were higher and ALT levels were lower in the HCV/HIV-co-infected group, whereas the inflammation and fibrosis METAVIR scores were comparable. The HCV/HIV-co-infected subjects had significantly higher triglyceride levels compared to the HCV-only infected subjects. All HIV-infected subjects had CD4 counts >200 cells/mm^3^ and 98% were receiving HAART.

### Quantification of PPARα mRNA expression by RT-PCR

**Figure 1:**
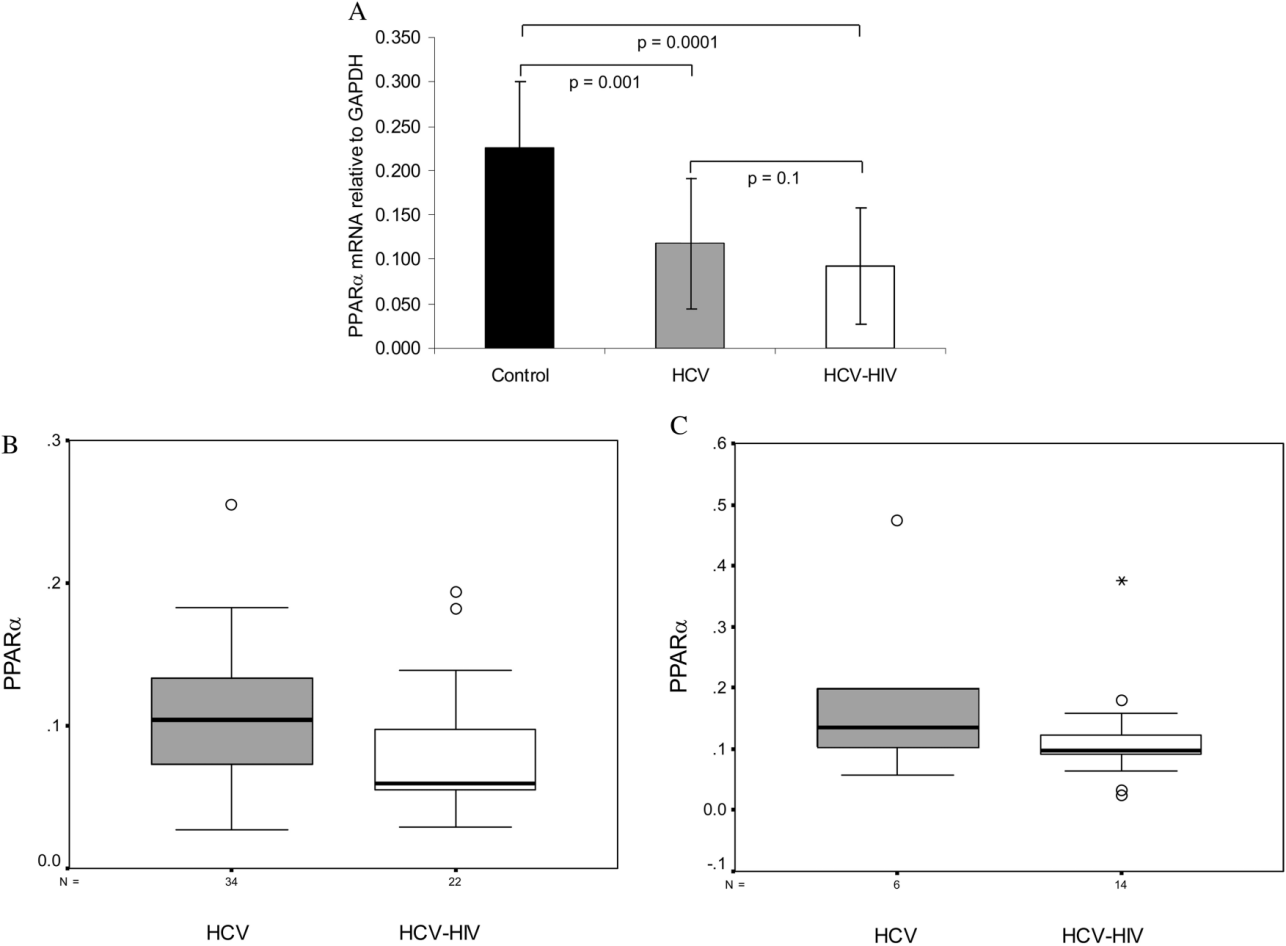
Quantification of liver peroxisome proliferator-activated receptor α mRNA expression by RT-PCR. (A) All patients: control (n=11), hepatitis C virus (n=40), and hepatitis C virus-HIV subjects (n=36) (error bars represent standard deviations). (B) Only non-black subjects: hepatitis C virus and hepatitis C virus-HIV subjects (*p*=0.02). (C) Only black subjects: hepatitis C virus and hepatitis C virus-HIV subjects (*p*=0.2).

Based on RT-PCR analyses of liver samples, controls had higher PPARα mRNA expression levels compared to both the HCV-only (*p*=0.001) and HCV/HIV-co-infected subjects (*p*=0.0001) (Figure 1A). The expression levels were comparable between the HCV-only and HCV/HIV-co-infected subjects (0.1178 *vs*. 0.0931; *p*=0.1). In both HCV-infected groups, the PPARα levels were higher among subjects from the United States (US) compared to non-US subjects (*p*=0.02). There were also differences in PPARα mRNA expression based on race, with black subjects having a significantly higher expression level compared to non-black subjects (0.1381 *vs*. 0.0946; *p*=0.02). Given these racial differences, additional analyses were performed to compare HCV-only infected and HCV/HIV-co-infected subjects stratified by race. A significantly lower level of PPARα mRNA expression was found among non-black subjects in the HCV/HIV-co-infected group compared to the HCV-only group (0.0769 *vs*. 0.1061; *p*=0.02) (Figure 1B), whereas there were no differences among black subjects (*p*=0.2) (Figure 1C). Other factors that were evaluated and found to have no association with PPARα gene expression included age, sex, BMI, HCV genotype, HCV RNA serum levels, DM, alcohol use, CD4 counts, serum HIV RNA levels and HAART therapy. In a multivariate linear regression model in which HIV status, black race, and country of origin were included, all three factors lost statistical significance. The pathology results demonstrated that moderate-to-severe inflammation was associated with lower PPARα mRNA expression among non-black, non-HIV subjects (0.0868 in METAVIR A2/A3 subjects *vs*. 0.1255 in A0/A1 subjects; *p*=0.01), but no associations were detected in the remaining groups.

### Quantification of PPARγ mRNA expression by RT-PCR

**Figure 2:**
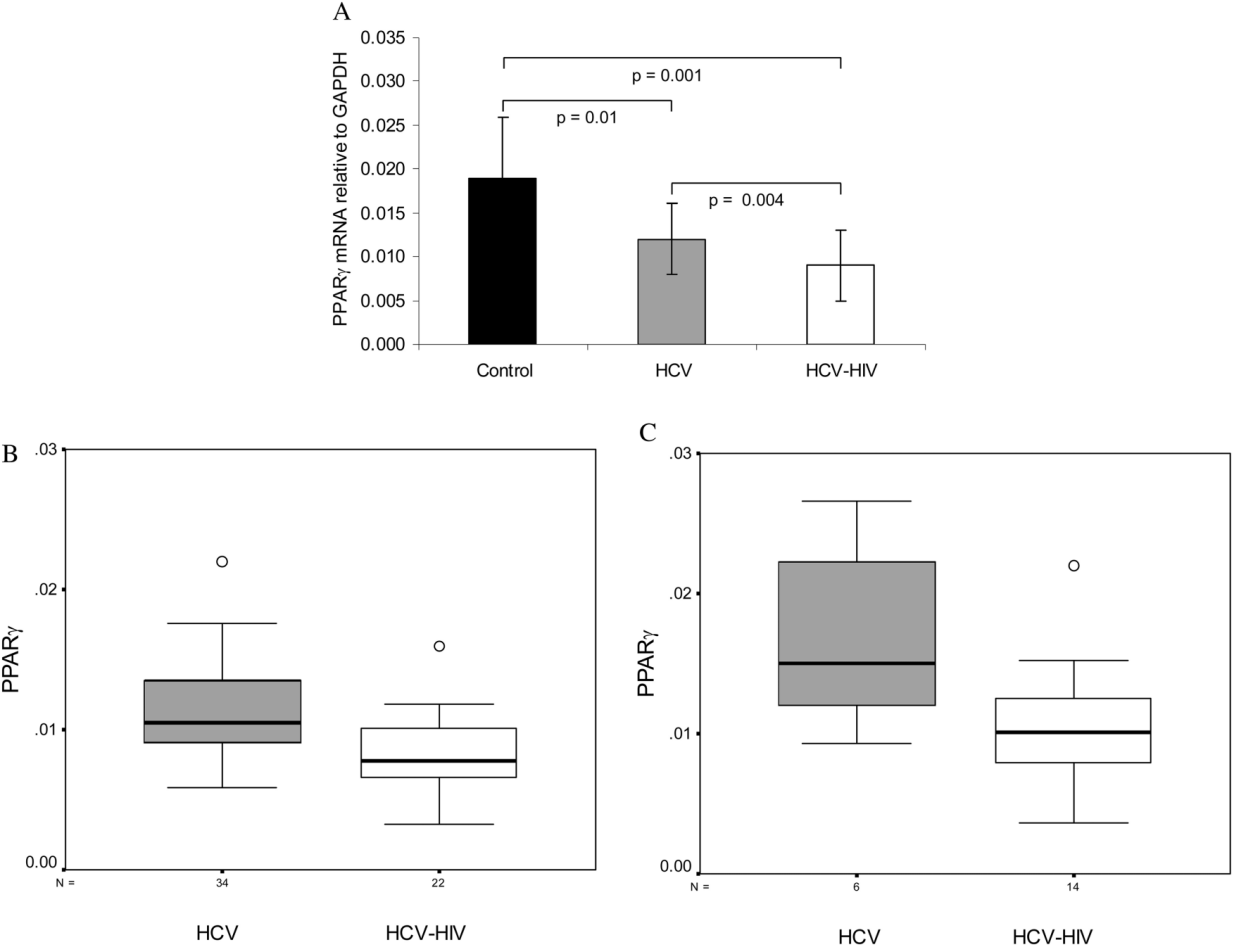
Quantification of liver peroxisome proliferator-activated receptor γ mRNA expression by RT-PCR. (A) All patients: control (n=11), hepatitis C virus (n=40), and hepatitis C virus-HIV subjects (n=36) (error bars represent standard deviations). (B) Only non-black subjects: hepatitis C virus and hepatitis C virus-HIV subjects (*p*=0.001). (C) Only black subjects: hepatitis C virus and hepatitis C virus-HIV subjects (*p*=0.03).

RT-PCR analysis of PPARγ mRNA in the liver samples indicate that the controls had a higher level of expression compared to both the HCV-only infected (*p*=0.01) and HCV/HIV-co-infected subjects (*p*=0.001) (Figure 2A). The expression levels were significantly higher in the HCV-only-infected subjects compared to the HCV-HIV-co-infected subjects (0.0120 *vs*. 0.0092; *p*=0.004). In both HCV-infected groups, PPARγ levels were higher among US subjects compared to non-US subjects (*p*=0.04). There were also differences in PPARγ expression based on race, with black subjects having a significantly higher expression level compared to non-black subjects (0.0101 *vs*. 0.0125; *p*=0.03). However, there was a significantly lower level of PPARγ mRNA expression in HCV-HIV-co-infected subjects compared to HCV-only subjects in both groups [non-black (*p*=0.001) (Figure 2B) and black subjects (*p*=0.03)] (Figure 2C). Other factors evaluated and found to have no association with PPARγ gene expression include age, sex, BMI, HCV genotype, HCV RNA serum levels, DM, alcohol use, CD4 counts, serum HIV RNA levels and HAART therapy (Table 2). In multivariate linear regression analyses, only HIV-co-infection (*p*=0.001) and non-black race (*p*=0.04) were independently associated with lower PPARγ expression (Table 2). The pathology findings demonstrated that more advanced fibrosis in liver biopsy was associated with lower hepatic PPARγ mRNA expression among non-black, non-HIV subjects (METAVIR score F2/F3/F4 0.0102 vs. METAVIR score F0/F1 0.0126; *p*=0.04). There was no association between PPARγ mRNA expression and steatosis in any group.

## DISCUSSION

Our results show that hepatic PPARα and PPARγ mRNA expression levels were reduced in the presence of HCV infection. Surprisingly, we observed marked differences by ethnicity; as a result, the power of the study to detect differences based on HIV status was diminished to a large extent, especially with regard to PPARα. To the best of our knowledge, this is the first report to examine or identify differences in PPARα and PPARγ liver expression by race. Our results suggest that genetic polymorphisms linked to ethnicity may explain this finding. Notably, PPAR has been implicated in the pathogenesis of hepatic steatosis, which has been reported to be less frequent among black subjects 8-10. Nevertheless, the non-black group is very heterogeneous because it includes Caucasians and mixed ethnicities, especially among subjects from Brazil. Because the liver samples were obtained from diverse races and different countries, we could not exclude the possibility that nutritional or other environmental factors might have had an effect on liver PPARα and/or PPARγ expression.

In line with previous reports, PPARα gene expression was impaired in the livers of HCV-infected subjects 3,4,11,12. Our data indicate that HIV infection also decreased PPARα gene expression, as revealed in non-black subjects. However, the effect of HIV was mitigated in black patients, suggesting that this group might be less vulnerable to the effects of the HIV virus on PPARα expression.

PPARα plays a pivotal anti-inflammatory role in hepatocytes 2,13. More severe liver inflammation in HCV-infected subjects with reduced PPARα expression in hepatocytes has been reported by some but not all authors 3,4,12. In our study, an inverse correlation between PPARα expression and necroinflammatory activity was only observed in the non-black HCV-only infected group based on METAVIR scores, a method that is likely inadequate for the detection of subtle trends in HIV-related fibrosis in HCV-infected patients. Larger studies with a more homogeneous population using a more precise method of inflammation measurement might shed additional light on this subject.

In contrast with prior reports, PPARγ gene expression was reduced in the livers of HCV-infected subjects compared to the controls 3,12. PPARγ expression in HCV-infected livers was further reduced by HIV co-infection. PPARγ is primarily expressed in hepatic stellate cells and its expression disappears when these cells undergo myofibroblastic differentiation and fibrogenesis 14. There appears to be a critical role for PPARγ in liver fibrogenesis, and treatment of hepatic stellate cells with synthetic PPARγ suppresses the fibrogenetic process 2,5. In our study, an inverse correlation was observed between PPARγ expression and more advanced fibrosis in the non-black HCV-only-infected group. The limitations of the present study can be overcome by measuring liver fibrosis using other methods, such as morphometric image analysis or quantification of fibrosis proteins and using a larger, more homogeneous population 9,15.

Our findings suggest that genetic polymorphisms may determine the expression of PPARα and PPARγ in the liver and that the impairment induced by the HCV and HIV viruses is mitigated in subjects of black ethnicity. Genetic studies are needed to determine why hepatic expression of these nuclear receptors differs by race. Despite the complexity introduced by race, PPARα and PPARγ hepatic expression were affected by HIV infection. PPARα expression is most robust in hepatocytes, although it is also expressed in other cell types. Hepatocytes have not been demonstrated to be infected by HIV, although HIV-1 has been proposed to be present in the liver and can promote fibrosis, most likely through indirect effects on hepatocytes 7,16. There is evidence from studies using cell lines that HIV may infect human stellate cells 17. The reduction in PPARγ expression, which is more abundant in stellate cells, might be due to direct infection by HIV. HIV may also affect PPARα and PPARγ expression via immune activation and the disruption of cellular functions.

Despite some limitations of our study, including lack of adequate samples to test protein expression, small sample size, ethnic and HCV genotype diversity of the subjects, and lack of a more refined measurement of liver inflammation and fibrosis, several findings are intriguing and deserve further attention. Additional studies are needed to elucidate the mechanisms underlying the decreased hepatic PPARα and PPARγ mRNA expression observed in our study. Thus, larger studies on HCV/HIV-co-infected individuals stratified by ethnicity and the inclusion of mRNA/protein expression of other genes associated with inflammation and fibrogenesis would shed light on these results. Further studies of genetic polymorphisms linked to ethnicity are also warranted to explain the differences in the expression of these PPARs.

### Funding source

This research was funded by Bristol-Myers Squibb through a grant within the Virology Fellows Research Training Program.

## AUTHOR CONTRIBUTIONS

Nunez M and High K conceived and designed the study. Shores N, Mendes-Correa MC, Maida I, Turner J and Babudieri S were responsible for the data acquisition. Nunez M, Shores N, High K, Turner J, Mendes-Correa MC and Maida I were responsible for the data analysis and interpretation. Nunez M, Shores N and Turner J were responsible for the manuscript drafting. Nunez M was responsible for the statistical analysis. Turner J was responsible for the technical and material support. Nunez M supervised the study. All authors have accepted the content of the manuscript.

## Figures and Tables

**Table 1:** Characteristics of the patients according to HIV status*.

	HCV-Only	HCV & HIV	*p-*value
N	40	36	---
Age	47 (28-69)	49 (36-68)	0.4
Male sex	29 (72.5)	24 (67)	0.4
Weight	82 (53-130)	75 (51-121)	0.2
BMI	28 (18-36)	26 (17-37)	0.3
Race			0.003
White	29 (72.5)	12 (33)	
Black	6 (15)	14 (39)	
Mixed/Hispanic	4 (10)	10 (28)	
Native American	1 (2.5)	0	
Country			<0.0001
Brazil	9 (22.5)	21 (58)	
Italy	12 (30)	0	
USA	19 (47.5)	15 (42)	
HCV genotype			0.5
1	34 (94)	30 (91)	
2	0	1 (3)	
3	1 (3)	2 (6)	
4	1 (3)	0	
HCV RNA, log_10_	5.82 (3.16-7.00)	6.17 (5.1-7.59)	0.04
ALT	100 (15-351)	62 (15-213)	0.02
AST	93 (20-1,018)	47 (16-103)	0.1
METAVIR fibrosis score			0.6
F0	4 (10)	6 (17)	
F1	14 (350	14 (39)	
F2	14 (35)	13 (36)	
F3	5 (12.5)	2 (5)	
F4	3 (7.5)	1 (3)	
METAVIR inflammation score			0.3
A0	1 (2.5)	5 (14)	
A1	17 (42.5)	15 (42)	
A2	17 (42.5)	11 (30)	
A3	5 (12.5)	5 (14)	
Steatosis			0.4
Unknown	9 (22.5)	7 (19.5)	
None	18 (45)	19 (53)	
Mild	6 (15)	8 (22)	
Moderate	4 (10)	1 (3)	
Severe	3 (7.5)	1 (3)	
CD4	---	540 (260-1049)	---
CD4%	---	27 (15-44)	---
HIV RNA	---	177 (<50-1450)	---
HAART			
No	---	3 (8)	---
AZT	---	11 (68)	---
Dideoxynucleosides	---	2 (6)	---
PI	---	20 (59)	---
DM	---	6 (17)	0.2
Current alcohol use	---	9 (26)	0.4
Cholesterol levels	169 (113-231)	159 (91-226)	0.4
Triglyceride levels	83 (37-2091	149 (55-428)	0.003

* Mean (range) unless otherwise stated.

BMI: body mass index; HCV: hepatitis C virus; HIV: human immunodeficiency virus; ALT: alanine aminotransferase; AST: aspartate aminotransferase; HAART: highly active antiretroviral therapy; AZT: azidothymidine; PI: protease inhibitor; DM: diabetes mellitus.

**Table 2:** PPARα mRNA expression: univariate and multivariate linear regression analyses.

	Univariate analyses Beta (95% CI); *p*-value	Multivariate Analysis Beta (95% CI); *p*-value
**HIV+**	−0.175 ((−0.057) − (−0.007)); 0.1	−0.225 ((−0.064) − (0.001)); *p*=0.06
**Age**	−0.086(−0.003 - 0.001); 0.4	
**Male sex**	−0.209 ((−0.067) − (0.003); 0.07	
**BMI**	0.067 (−0.003 - 0.005); 0.6	
**Black race**	**0.272 (0.008 – 0.079); 0.02**	0.225 (−0.008 - 0.080); 0.1
**United States**	**0.333 (0.016 - 0.078); 0.003**	0.197((−0.010) − 0.065)); 0.1
**HCV-1 genotype**	0.216 ((−0.006) − (0.126); 0.07	
**HCV RNA**	−0.144 (0.000 - 0.000); 0.3	
**METAVIR F3/F4**	−0.112((−0.068) − (0.024); 0.3	
**METAVIR A2/A3**	0.063 ((−0.024) – (0.041)); 0.6	
**Steatosis (yes *vs*. no)**	−0.105 ((−0.057) – (0.024)); 0.4	
**DM**	0.054 ((−0.04) – (0.063)); 0.6	
**Alcohol use**	0.120 ((−0.017) − (0.055)); 0.3	
**CD4 counts**	−0.235 (0.000 - 0.000); 0.3	
**HIV RNA levels**	0.112 (0.000 - 0.000); 0.7	
**HAART regimen**		
** AZT**	−0.337 ((−0.029) – (0.000)); 0.051	
** Dideoxynucleosides**	0.029 ((−0.090) – (0.016)); 0.9	
** PI**	−0.038 ((−0.052) − 0.042); 0.8	

BMI: body mass index; HCV: hepatitis C virus; HAART: highly active antiretroviral therapy; AZT: azidothymidine; PI: protease inhibitor; DM: diabetes mellitus.

**Table 3:** PPARγ mRNA expression: univariate and multivariate linear regression analyses.

	Univariate analyses Beta (95% CI); *p*-value	Multivariate Analysis Beta (95% CI); *p*-value
**HIV+**	**−0.330 ((−0.005) − (−0.001)); 0.004**	**−0.392 ((−0.005) − (−0.001)); p=.001**
**Age**	−0.142 (0.000 - 0.000); 0.2	
**Male sex**	−0.046 ((−0.003) − (0.002); 0.7	
**BMI**	0.085 (0.000 - 0.000); 0.9	
**Black race**	**0.25 (0.000 – 0.005); 0.03**	**0.266 (0.000 - 0.005); 0.04**
**United States**	**0.333 (0.001 - 0.005); 0.003**	0.166 ((−0.001) − 0.004)); 0.2
**HCV-1 genotype**	0.133((−0.002) − (0.006); 0.3	
**HCV RNA**	−0.109 (0.000 - 0.000); 0.4	
**METAVIR F3/F4**	−0.08 ((−0.004) − (0.002); 0.5	
**METAVIR A2/A3**	0.128 ((−0.001) – (0.003)); 0.9	
**Steatosis (yes *vs*. no)**	−0.051 ((−0.003) – (0.002)); 0.7	
**Diabetes**	0.056 ((−0.002) – (0.004)); 0.6	
**Alcohol use**	0.065 ((−0.002) − (0.003)); 0.5	
**CD4 counts**	−0.07 (0.000 - 0.000); 0.7	
**HIV RNA levels**	−0.017 (0.000 - 0.000); 0.9	
**HAART regimen**		
** AZT**	−0.297 ((−0.005) – (0.000)); 0.09	
** Dideoxynucleosides**	−0.022 ((−0.006) – (0.005)); 0.9	
** PI**	−0.06 ((−0.003) − 0.002); 0.7	

BMI: body mass index; HCV: hepatitis C virus; HAART: highly active antiretroviral therapy; AZT: azidothymidine; PI: protease inhibitor; DM: diabetes mellitus.
